# MINDIN Exerts Protumorigenic Actions on Primary Prostate Tumors via Downregulation of the Scaffold Protein NHERF-1

**DOI:** 10.3390/cancers13030436

**Published:** 2021-01-24

**Authors:** Luis Álvarez-Carrión, Irene Gutiérrez-Rojas, María Rosario Rodríguez-Ramos, Juan A. Ardura, Verónica Alonso

**Affiliations:** 1 Bone Physiopathology Laboratory, Applied Molecular Medicine Institute (IMMA), Universidad San Pablo-CEU, CEU Universities, Campus Monteprincipe, 28925 Alcorcón, Spain; luis.alvarezcarrion@ceu.es (L.Á.-C.); Irene.gutierrezrojas@ceu.es (I.G.-R.); mrrodram@ceu.es (M.R.R.-R.); 2 Departamento de Ciencias Médicas Básicas, Facultad de Medicina, Universidad San Pablo-CEU, CEU Universities, Campus Monteprincipe, 28925 Alcorcón, Spain

**Keywords:** NHERF-1, MINDIN, prostate cancer, bone, metastases, migration, osteomimicry

## Abstract

**Simple Summary:**

Prostate cancer is one of the leading causes of death among men worldwide. Advanced prostate cancer is an incurable disease whose mechanisms of action are still not fully understood. Secretion of the matrix protein MINDIN has been associated with prostate tumor development towards advanced prostate cancer. We aimed to study the mechanisms whereby MINDIN promotes prostate cancer progression. Evaluation of human and mouse prostate cancer samples showed increased MINDIN expression associated with decreased expression of the adaptor protein Na+/H+ exchanger regulatory factor 1 (NHERF-1). We found that NHERF-1 was downregulated by MINDIN in prostate cancer, causing an increase in tumor cell migration and proliferation. These observations point to NHERF-1 as a key modulator of MINDIN actions on prostate cancer progression and suggest that both proteins could be potential targets for the development of future prostate cancer therapies.

**Abstract:**

Advanced prostate cancer preferential metastasis to bone is associated with osteomimicry. MINDIN is a secreted matrix protein upregulated in prostate tumors that overexpresses bone-related genes during prostate cancer progression. Na+/H+ exchanger regulatory factor (NHERF-1) is a scaffold protein that has been involved both in tumor regulation and osteogenesis. We hypothesize that NHERF-1 modulation is a mechanism used by MINDIN to promote prostate cancer progression. We analyzed the expression of NHERF-1 and MINDIN in human prostate samples and in a premetastatic prostate cancer mouse model, based on the implantation of prostate adenocarcinoma TRAMP-C1 (transgenic adenocarcinoma of the mouse prostate) cells in immunocompetent C57BL/6 mice. The relationship between NHERF-1 and MINDIN and their effects on cell proliferation, migration, survival and osteomimicry were evaluated. Upregulation of MINDIN and downregulation of NHERF-1 expression were observed both in human prostate cancer samples and in the TRAMP-C1 model. MINDIN silencing restored NHERF-1 expression to control levels in the mouse model. Stimulation with MINDIN reduced NHERF-1 expression and triggered its mobilization from the plasma membrane to the cytoplasm in TRAMP-C1 cells. MINDIN-dependent downregulation of NHERF-1 promoted tumor cell migration and proliferation without affecting osteomimicry and adhesion. We propose that MINDIN downregulates NHERF-1 expression leading to promotion of processes involved in prostate cancer progression.

## 1. Introduction

Prostate cancer is the second most common cancer among men after lung cancer and is one of the leading causes of cancer mortality and morbidity globally [1]. Advanced solid tumors from prostate, breast, melanoma or lung cancers usually metastasize to the bone microenvironment. Preferential metastasis to bone—a process known as bone tropism—has a relative incidence of 65–75% in prostate tumors and no current treatment [2,3,4,5]. In order for primary tumor cells to reach bone, a series of features to induce migration and successful “homing” to the skeleton must be acquired by these cells [6]. Some studies suggest that bone tropism of prostate cancer cells is caused by the acquisition of bone-related or osteomimicry features. Thus, prostate cancer cells ectopically express markers of bone, such as the bone formation transcription factor Runx2 or the bone resorption factor Receptor Activator of Nuclear Factor Kappa-β-Ligand (RANK-L) and behave as bone cells to adapt and grow in the bone microenvironment [6,7,8,9]. In addition, the capability of primary tumor cells to colonize bone is also associated with activation of bone cells. Cancer cells promote the secretion of growth factors and cytokines by osteoblasts (bone formation cells), osteoclasts (bone degradation or resorption cells) and osteocytes (bone monitoring cells). These factors, in turn, enhance tumor growth, metastatic cell proliferation and signaling [10,11]. The resulting “vicious cycle” of bone metastases changes bone physiology and triggers uncoupled bone remodeling [12,13,14]. Since this process has not been characterized extensively, the study of factors that mediate prostate cancer bone metastasis could lead to the identification of potential diagnosis biomarkers or therapeutic targets.

MINDIN, also known as SPONDIN-2, is a secreted extracellular matrix protein that belongs to the thrombospondin superfamily of proteins that contain type 1 repeat (TSR-1) domains. This protein has recently been suggested as a specific diagnosis biomarker of prostate cancer progression [15,16] and was revealed to be overexpressed in prostate cancer patients with bone metastases [17]. Furthermore, our group has recently described that MINDIN increases osteomimicry and tumor progression markers in primary prostate tumors [18] and induces premetastatic changes in bone [19]. However, the mechanisms whereby MINDIN promotes prostate tumor progression are still ill-defined.

Na^+^/H^+^ exchanger regulatory factor 1 (NHERF-1), otherwise ezrin-radixin-moesin-binding phosphoprotein 50 (EBP50), is a scaffold protein that possesses two type 1 tandem PSD-95/Discs Large/ZO-1 (PDZ) domains and a carboxyl-terminal ezrin-binding domain (EBD) [20,21,22]. PDZ domains function as protein-protein interaction modules and are able to interact with multiple PDZ recognition motif containing proteins including multiple transporters, as well as G protein-coupled receptors and cytosolic signaling effector proteins [23,24,25]. The EBD domain interacts with the N-terminal domain of ezrin, leading to indirect attachment to the cytoskeleton [26,27]. NHERF-1 may homo- or heterodimerize and is enriched in tissues possessing a polarized epithelia such as prostate epithelium [22]. NHERF-1 is predominantly found in brush-border membranes, although it is present in basolateral membranes too [28,29]. NHERF-1 can recruit membrane receptors and transporters as well as cytoplasmic signaling proteins and transcriptional coactivators to regulate many processes such as cell proliferation, survival, apoptosis, migration and invasion [30,31,32,33,34,35]. NHERF-1 is capable of recruiting the tumor suppressor PTEN to inactivate PI3K/AKT proproliferative and prosurvival signaling pathway in glioblastoma multiforme [36,37,38]. Stabilization of β-catenin at cellular junctions in murine embryonic fibroblast models is also potentially induced by NHERF-1 [39]. Although these features are indicative of a tumor suppressor function, NHERF-1 has been involved in both tumor progression and inhibition [20,32,33,34,36,37,38,39,40,41,42,43,44,45,46]. An immunohistochemical study in prostatic cancer has revealed decreased NHERF-1 presence in primary and metastatic prostatic adenocarcinoma samples [20]. Nevertheless, the mechanisms that trigger NHERF-1 decreased levels in prostate cancer and the cellular processes affected in prostate cancer cells by NHERF-1 downregulation are unknown. In this regard, NHERF-1 has shown to regulate bone transcription factors and bone formation processes in osteoblasts [47]. We have recently described that MINDIN is overexpressed in prostate cancer associated with increased expression of bone-related proteins in the prostate [18]. Given that MINDIN induces osteomimicry in prostate cancer cells [18] and regulates bone processes during premetastatic development [19], we hypothesize that regulation of NHERF-1 is one of the mechanisms used by MINDIN during prostate progression towards a bone metastatic phenotype.

## 2. Results

### 2.1. MINDIN and NHERF-1 Show Opposite Patterns of Expression in Human Prostate Tumors

**Figure 1:**
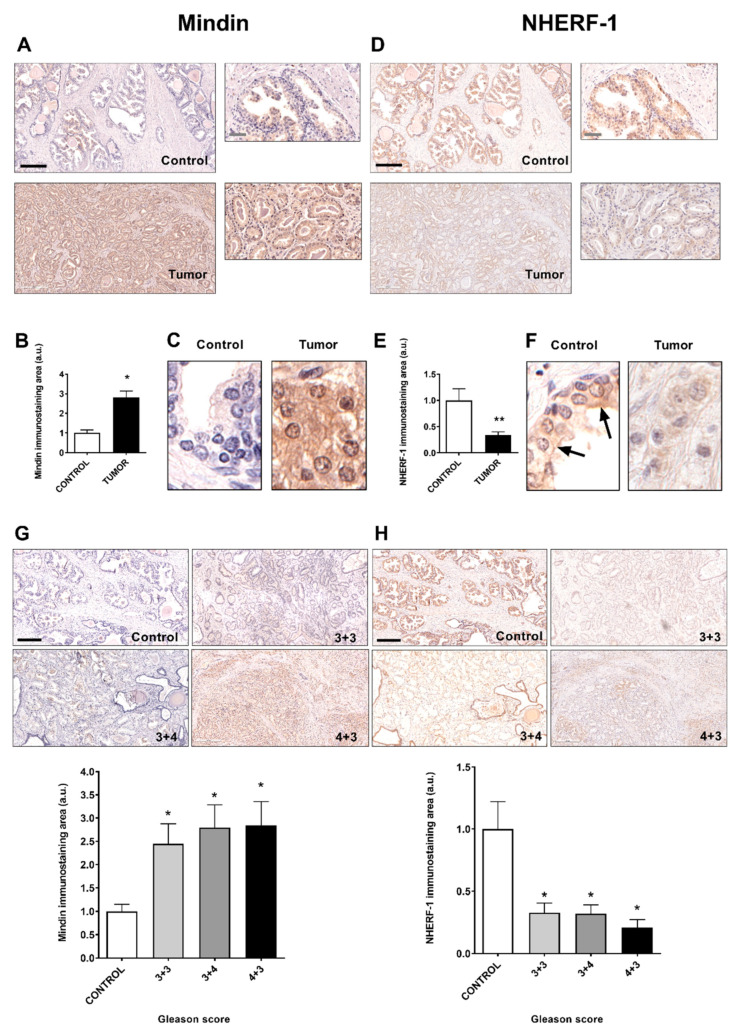
Human prostate tumors classified according to Gleason score show increased levels of MINDIN and decreased Na+/H+ exchanger regulatory factor 1 (NHERF-1) immunolabeling. Evaluation of MINDIN and NHERF-1 immunolabeling levels was performed in human control and prostate tumor samples by immunohistochemistry. Representative images, bar graph and detailed images of MINDIN (**A**–**C**) and NHERF-1 (**D**–**F**) immunostaining levels in control and tumor samples are shown. Representative images and evaluation of MINDIN (**G**) and NHERF-1 (**H**) immunostaining levels according to Gleason score classification are shown. Black arrows indicate apical NHERF-1 immunostaining. Black and grey scale bars represent 500 and 100 µm, respectively. Magnification ×100 (**A**,**D**,**G** and **H**), ×400 (**A** and **D** details) and ×600 (**C** and **F**). Experimental values show mean ± Standard Error of the Mean (SEM). *n* = 12–18 samples/group. * *p* < 0.05 vs. control; ** *p* < 0.01 vs. control samples.

We have recently described that MINDIN is overexpressed in human prostate cancer cells associated with changes in osteomimicry markers [18], and regulates bone processes prior to metastatic development [19]. To test whether there is an association between MINDIN and the osteogenic-related factor NHERF-1 in prostate cancer, we first analyzed by immunohistochemistry the expression and subcellular localization of MINDIN and NHERF-1 in human prostate control and tumor samples. Patients were classified according to Gleason score, D`Amico risk, presence of perineural invasion and positive surgical margins, TNM stage and age. We observed that MINDIN immunolabeling was increased in prostate tumors compared to control samples (Figure 1A,B). In addition, MINDIN was found in the plasma membrane, cytoplasm and extracellular space of prostate cancer samples, whereas MINDIN immunostaining was negligible in control samples (Figure 1C). In contrast, NHERF1 immunolabeling was decreased in prostate cancer compared to control samples (Figure 1D,E). Furthermore, NHERF-1 was found both at the apical plasma membrane and cytoplasm in control prostate samples, whereas the scarce presence of NHERF-1 showed in prostate tumors was predominantly cytoplasmic (Figure 1F). Regarding the Gleason score, MINDIN and NHERF-1 immunolabeling were increased or decreased in tumors with Gleason grades 3+3, 3+4 and 4+3 compared to control samples, respectively (Figure 1G,H).

**Figure 2:**
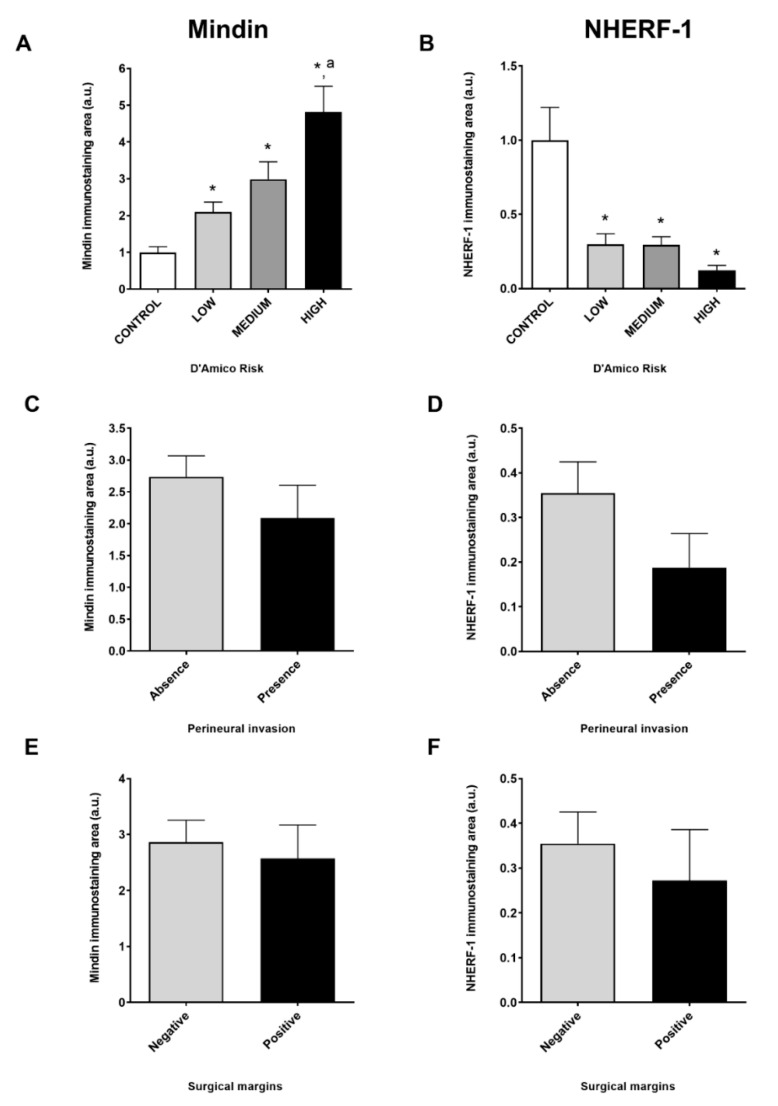
Human prostate tumors classified according to D`Amico risk show increased levels of MINDIN and decreased NHERF-1 immunolabeling. Evaluation of MINDIN and NHERF-1 immunolabeling levels was performed in human control and prostate tumor samples by immunohistochemistry. Bar graphs representing MINDIN (**A**,**C**,**E**) and NHERF-1 (**B**,**D**,**F**) immunostaining levels in control and tumor samples classified according to D’Amico Risk (**A** and **B**), perineural invasion (**C** and **D**) and surgical margins (**E** and **F**) are shown. Experimental values show mean ± SEM. *n* = 12–18 samples/group. * *p* < 0.05 vs. control; ^a^ *p* < 0.05 vs. low and medium D`Amico risk samples.

D`Amico risk classification showed that MINDIN immunolabeling was increased in tumors with high risk, followed by medium and low risk (Figure 2A). Unlike MINDIN, NHERF-1 immunolabeling was decreased in tumors with low, medium and high D`Amico risks (Figure 2B). We did not find differences in MINDIN or NHERF-1 immunolabeling regarding the presence or absence of perineural invasion (Figure 2C,D), negative or positive surgical margins (Figure 2E,F) and TNM stage or age. Additionally, tumor specimens showed upregulated expression of bone-related-factors such as osterix (control = 0.47 ± 0.14 vs. tumor = 3.52 ± 0.74 arbitrary units (a.u.)) and RANKL (control = 0.0016 ± 0.0004 vs. tumor = 0.0233 ± 0.0104 a.u.) compared to control samples. These results suggest that increased levels of MINDIN in prostate tumors are associated with a decrease in NHERF-1 immunolabeling.

### 2.2. MINDIN Reduces NHERF-1 Expression and Triggers Its Mobilization to the Cytoplasm in Prostate Tumor Cells

**Figure 3:**
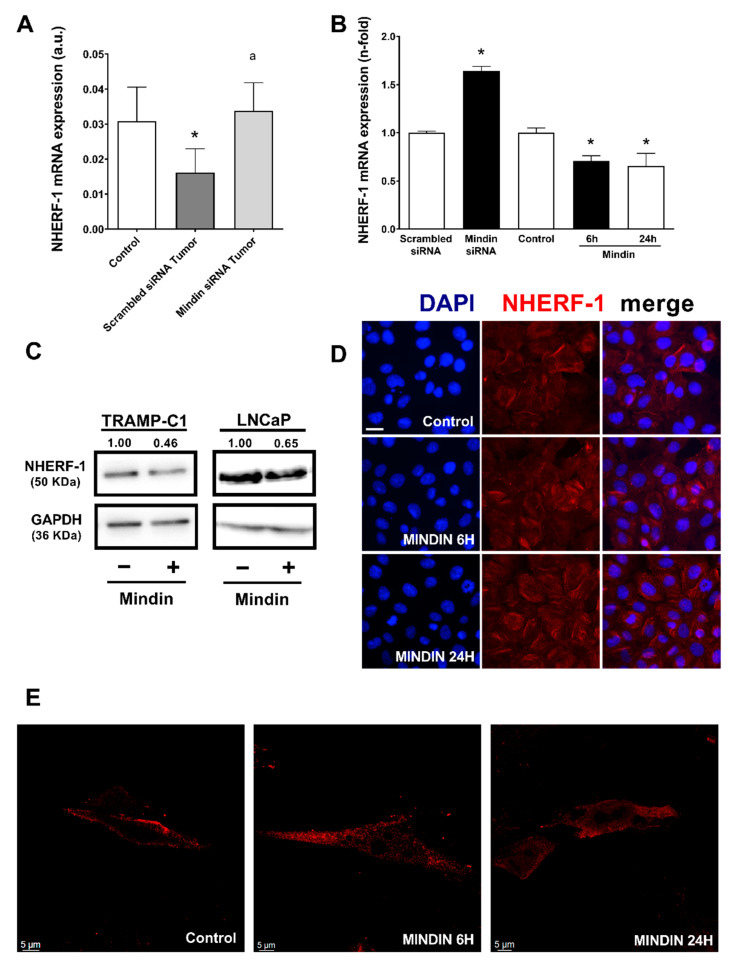
MINDIN decreases NHERF-1 expression in mouse prostate tumors and in TRAMP-C1 prostate adenocarcinoma cells. (**A**) Expression of NHERF-1 mRNA levels was evaluated in control and in TRAMP-C1-induced prostate tumor samples by real time PCR. Prostate tumors were induced by orthotopical injection with scrambled siRNA- or MINDIN siRNA silenced TRAMP-C1 cells as described in Materials and Methods. *n* = 7–10 samples/group. Groups: control (no prostate tumor); scrambled siRNA tumor (prostate tumors induced by scrambled silenced TRAMP-C1 cells); MINDIN siRNA tumor (prostate tumors induced by MINDIN silenced TRAMP-C1 cells). (**B**) TRAMP-C1 cells were silenced for 24 h with either scrambled or MINDIN siRNAs or stimulated with 5 ng/mL MINDIN for 6 h and 24 h. NHERF-1 mRNA levels were tested by real time PCR. (**C**) TRAMP-C1 and LNCaP prostate cancer cells were stimulated with 5 ng/mL MINDIN for 24 h. NHERF-1 protein levels were assessed by Western blot. Representative Western blot autoradiograms and densitometric values are shown. The corresponding full uncropped Western Blot is shown in Figure S1 * *p* < 0.05 vs. control or scrambled siRNA; ^a^ *p* < 0.05 vs. scrambled siRNA tumor. (**D**) Representative epifluorescence images depicting DAPI staining and NHERF-1 immunostaining in TRAMP-C1 prostate cancer cells stimulated with 5 ng/mL MINDIN for 6 h or 24 h or not (control). Scale bar, 20 µm. (**E**) Representative confocal images depicting NHERF-1 immunostaining in TRAMP-C1 prostate cancer cells stimulated with 5 ng/mL MINDIN for 6 or 24 h or not (control). Scale bar, 5 µm.

Our group has previously described that some osteomimicry features are promoted by MINDIN in primary prostate tumors of a mouse model in which TRAMP-C1 adenocarcinoma cells were orthotopically injected into C57BL/6 male mice to induce prostate tumors [18]. In the present manuscript, we used this mouse model to test whether MINDIN could regulate NHERF-1 expression in prostate cancer. Primary prostate tumors of this model showed significant upregulated mRNA levels of MINDIN compared to controls (control = 0.0001 ± 0.0001 vs. scrambled siRNA tumor = 0.0054 ± 0.0020 a.u.), whereas MINDIN silencing in prostate tumors decreased the levels of this protein (scrambled siRNA tumor = 0.0054 ± 0.0020 vs. MINDIN siRNA tumor = 0.0002 ± 0.0001 a.u.). Primary tumors that exhibited increased levels of MINDIN showed decreased expression of NHERF-1 compared to control prostates without tumors (Figure 3A). Furthermore, we observed that MINDIN silencing increased the expression of NHERF-1 in primary prostate tumors (Figure 3A).

Following, we aimed to test the effects of MINDIN on the expression and subcellular localization of NHERF-1 in vitro. Stimulation with MINDIN caused a decrease in NHERF-1 mRNA and protein expression in TRAMP-C1 cells (Figure 3B,C). Similar results were observed in human adenocarcinoma LNCaP cells (Figure 3C). Moreover, silencing of MINDIN increased the expression of NHERF-1 in TRAMP-C1 cells (Figure 3B). An 85% efficiency of MINDIN silencing was corroborated by real time PCR.

MINDIN not only decreased NHERF-1 expression but also induced the relocalization of this protein from the plasma membrane of TRAMP-C1 cells to the cytoplasm of these cells (Figure 3D,E).

Altogether, these data suggest that increased levels of MINDIN alter the functionality of NHERF-1 by decreasing its expression and by triggering NHERF-1 mobilization from the plasma membrane to the cytoplasm in prostate adenocarcinoma cells.

### 2.3. Downregulation of NHERF-1 Expression Mediates MINDIN Effects on Prostate Adenocarcinoma Cell Migration and Proliferation

We next evaluated the actions of NHERF-1 on cellular processes that have previously been described to be triggered by MINDIN in prostate tumor cells and are involved in tumor progression, such as cell migration, proliferation and osteomimicry features [18].

**Figure 4:**
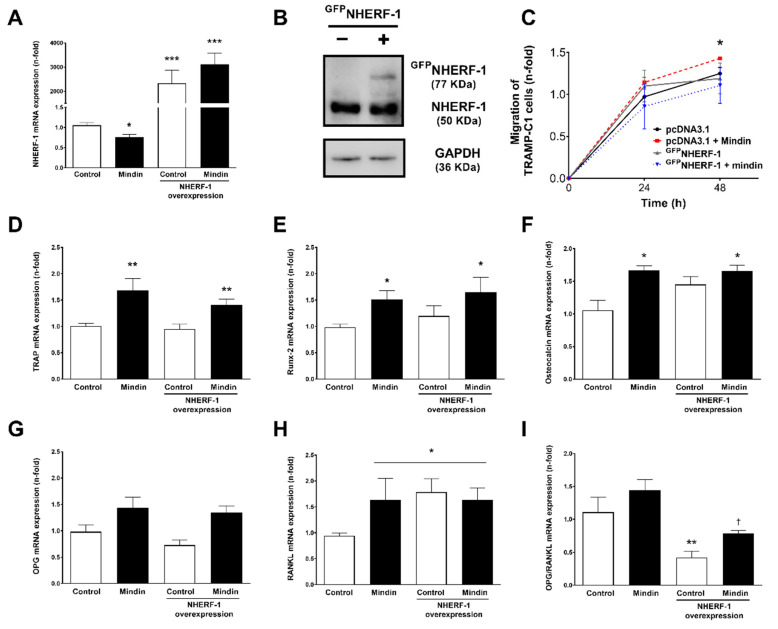
NHERF-1 overexpression inhibits MINDIN-induced migration of TRAMP-C1 prostate adenocarcinoma cells. Overexpression of NHERF-1 in TRAMP-C1 cells was achieved by transient transfection using a ^GFP^NHERF-1 plasmid construct. NHERF-1 mRNA (**A**) and protein (**B**) expression were evaluated by quantitative PCR and Western blot in ^GFP^NHERF-1-transfected cells, respectively. The corresponding uncropped Western Blot is shown in Figure S2. (**C**) Cell migration was assessed in TRAMP-C1 prostate cancer cells transfected with a pcDNA 3.1 empty vector or with ^GFP^NHERF-1 plasmid construct and stimulated or not with 5 ng/mL MINDIN as described in Materials and Methods. Evaluation of Tartrate-Resistant Acid Phosphatase (TRAP) (**D**), Runx2 (**E**), Osteocalcin (**F**), OPG (**G**) and RANK-L (**H**) mRNA expression and the OPG/RANK-L mRNA ratio (**I**) was assessed by real time PCR. Experimental values are mean ± SEM from 3 independent experiments. * *p* < 0.05 vs. control; ** *p* < 0.01 vs. control; *** *p* < 0.01 vs. control: † *p* < 0.05 vs. MINDIN.

To test the putative actions of NHERF-1 on the aforementioned MINDIN-induced cell processes, an approach based on GFP-tagged NHERF-1 upregulation (^GFP^NHERF) by a plasmid expression vector was used. Overexpression of NHERF-1 was confirmed by checking mRNA and protein expression (Figure 4A,B) and positive GFP fluorescence comparing to transfection with a pcDNA3.1 empty vector. We observed in ^GFP^NHERF-transfected cells that MINDIN-induced cell migration was reduced by NHERF-1 overexpression (Figure 4C). Regarding the expression of bone-related genes, MINDIN-dependent increased levels of TRAP, Runx2 and osteocalcin were unaffected by ^GFP^NHERF-1 overexpression (Figure 4D–F). While stimulation with MINDIN or NHERF-1 overexpression caused no significant effects on OPG mRNA levels (Figure 4G), both MINDIN and NHERF-1 triggered upregulation of RANKL levels (Figure 4H), leading to decreased OPG/RANKL ratios compared to control cells (pcDNA3.1 plasmid-transfected cells) (Figure 4I).

**Figure 5:**
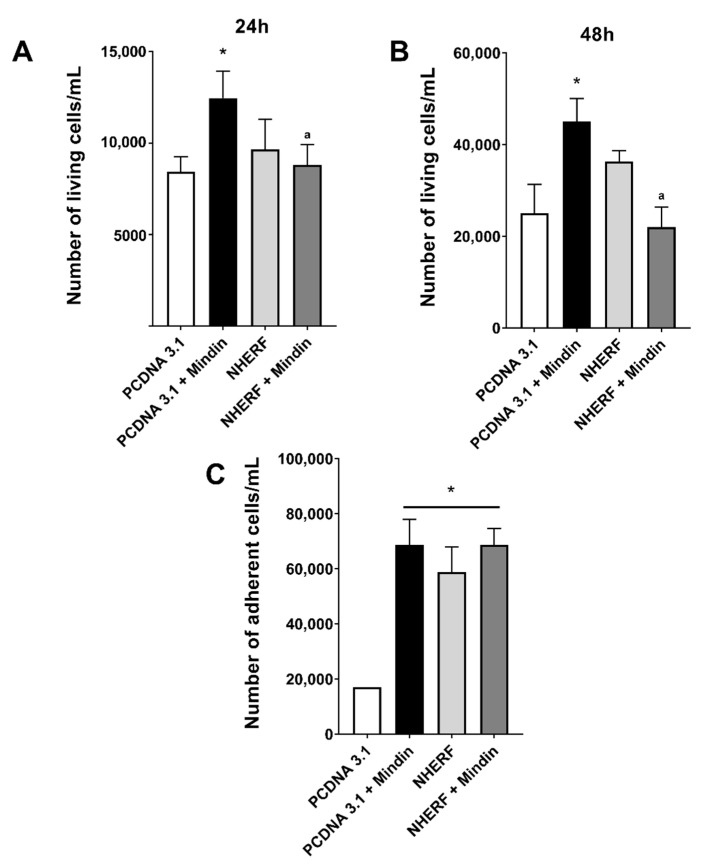
NHERF-1 overexpression inhibits MINDIN-induced proliferation of TRAMP-C1 prostate adenocarcinoma cells. Overexpression of NHERF-1 in TRAMP-C1 cells was achieved by transient transfection using a ^GFP^NHERF-1 plasmid construct. Cell proliferation after 24 (**A**) or 48 h (**B**) of 5 ng/mL MINDIN stimulation in pcDNA 3.1 empty vector- or ^GFP^NHERF-1-transfected TRAMP-C1 cells was assessed by trypan blue exclusion assay as described in materials and methods. (**C**) The number of TRAMP-C1 cells adhered to colagenized plate surfaces was evaluated in TRAMP-C1 prostate cancer cells transfected with a pcDNA 3.1 empty vector or with ^GFP^NHERF-1 plasmid construct and stimulated or not with 5 ng/mL MINDIN for 24 h as described in Materials and Methods. Experimental values represent mean ± SEM from 3 independent experiments. * *p* < 0.05 vs. PCDNA 3.1 control; ^a^ *p* < 0.05 vs. PCDNA 3.1 + MINDIN.

Similarly, NHERF-1 overexpression abolished the proliferative effects induced by MINDIN after 24 and 48 h of stimulation with this peptide (Figure 5A,B). However, an adhesion assay revealed that even though MINDIN induced an increase in cell adherence, NHERF-1 overexpression was inefficient inhibiting MINDIN-dependent cell adhesion (Figure 5C).

Our data indicate that NHERF-1 downregulation mediates MINDIN-induced prostate cancer cell migration and proliferation and modulates RANKL expression. In contrast, NHERF-1 does not affect MINDIN-dependent effects on other osteomimicry factors or on prostate cancer cell adhesion.

Altogether, these observations suggest that NHERF-1 mediates key cellular events induced by MINDIN during prostate cancer progression.

## 3. Discussion

Bone metastases derived from advanced solid tumors such as prostate cancer are painful, difficult to cure and have a poor survival prognosis [2,3,4,5]. The complex process required to produce bone metastatic lesions is based on tumor cell phenotypic plasticity, which enables the acquisition of prostate cancer prometastatic phenotypes such as osteomimicry. Although this process has been extensively studied, it is still not fully understood how different proteins interact to orchestrate prostate tumor progression. Here, we describe the key role of the osteogenic factor NHERF1 as a mediator of the protumorigenic actions of MINDIN during prostate cancer progression.

Most studies analyzing serum levels of MINDIN in a prostate cancer context have reported increased concentrations of MINDIN in the serum of prostate cancer patients compared with healthy subjects [15,16,17,48]. Supporting the notion of MINDIN upregulation in the serum of prostate cancer patients, some studies of other groups and ours have shown overexpression of MINDIN in prostate cancer compared to control tissue samples or cells [15,16,18,19,48,49]. In contrast, decreased levels of MINDIN have been described in the sera of patients with other types of cancer (i.e., gastric, esophageal, colon, lung and breast cancer) [50]. Altogether, these observations might suggest that MINDIN increases in the serum of patients with certain types of cancers such as prostate or ovarian cancer [51], while decreasing in other types. However, a decrease in serum levels of MINDIN in prostate cancer patients compared to control subjects has also been reported in two independent studies [52,53]. These discrepancies could be attributable to different assay methodologies but are more likely caused by differences in the selection of control subjects. In this regard, Jokerst et al. [52] used samples from benign prostatic hypertrophy patients as controls, raising the possibility that levels of MINDIN could increase during prostatic hypertrophy above values seen in either normal or prostate cancer.

Interestingly, MINDIN expression has been reported to be higher in specimens from patients with more aggressive prostate cancer and worse prognosis, with Gleason score sums of 7–8, and in those with bone metastases [16,17]. Moreover, the highest MINDIN levels have been found in prostate cancer individuals with bone metastasis, followed by individuals with hyperplasia and without metastatic disease [17]. In this regard, our data also show that MINDIN immunostaining is increased in samples with high Gleason scores and D`amico risk values, supporting the role of MINDIN as a putative biomarker for prostate cancer. Although MINDIN has been defined as an extracellular matrix protein, our results and previous reports [16] show strong cytoplasmic in addition to extracellular immunostaining. As an extracellular matrix protein, MINDIN has been associated with opsonin roles for macrophages as part of the innate immune system [54]. Cytoplasmic distribution of MINDIN is likely to have a different but still unknown biological role. We have recently described the role of MINDIN as a promoter of prostate tumor progression [18] and as an inductor of premetastatic changes in bone [19] via activation of ERK 1/2 and β-catenin pathways, respectively. Actions of MINDIN on the bone microenvironment are probably due to extracellular interactions of the secreted MINDIN pool with bone cells, but effects on proliferation and osteomimicry of primary prostate tumor cells could be caused by extracellular but also cytoplasmic pools of MINDIN. Further studies would be required to unravel these roles.

Regarding NHERF-1, this scaffolding protein has been involved in both tumor progression and inhibition in different types of cancer [20,32,33,34,36,37,38,39,40,41,42,43,44,45,46]. Our data show a decrease in NHERF-1 immunolabeling in primary human prostate tumors compared to control samples. Supporting these observations, a previous study revealed that the average immunostaining intensities of NHERF-1 are lowest in the specimens of prostate cancer and in those with bone metastases compared to control samples [20]. In particular, metastastic samples have shown significantly lower staining than all other samples and tissue types, indicating that loss of NHERF-1 expression may play a critical role in prostate cancer metastasis [20]. Even though we have observed a slight decrease in NHERF-1 immunostaining in specimens with high compared to low Gleason scores and D`Amico risks, these differences were not significant.

Our assessment of NHERF-1 localization in subcellular compartments shows that NHERF-1 is present at the apical cell membrane and cytoplasmic compartments in control samples whereas tumor samples show decreased levels and cytoplasmic expression of NHERF-1. NHERF-1 is usually highly expressed and localized at the cell membranes of physiological epithelial tissues along with the cortical actin cytoskeleton [55,56,57,58]. Furthermore, it has been observed that epithelial cell polarity is lost when NHERF-1 is not expressed at the apical plasma membrane [42] because apical localization of NHERF-1 is required to maintain epithelial integrity [59]. In this regard, NHERF-1 loss at the cell membrane in tumor specimens could be associated with the acquisition of features that are typical of epithelial–mesenchymal transition (EMT), a process whereby cells lose their epithelial characteristics such as polarity and cell–cell contact and acquire mesenchymal features during the progression of several tumors including prostate cancer [60]. In fact, previous studies have associated NHERF-1 loss of physiological apical membrane distribution to cytoplasmic expression with EMT and increased cell migration and invasion in other types of cancer [42]. The mechanisms that trigger NHERF-1 downregulation and mobilization from the plasma membrane have not previously been described in prostate cancer. Our in vivo and in vitro results show that NHERF-1 downregulation is induced by MINDIN in prostate tumor cells. We have recently described that MINDIN activates the Wnt/β-catenin signaling pathway, being a potential pivotal mechanism in prostate tumor progression and metastasis to bone [19]. Interestingly, Wnt/β-catenin activation has been associated with EMT [60] and NHERF-1 has been found to regulate the Wnt/β-catenin pathway: NHERF-1 has been shown to be required to maintain a fraction of β-catenin at the cortical submembrane compartment under physiological conditions [39]. In contrast, NHERF-1 accumulation has been described both in the cytoplasm and nuclei of human hepatocellular carcinoma cells, where NHERF-1 localization correlates with that of nuclear β-catenin, suggesting a functional interaction between these two proteins [41]. The presence of NHERF-1 in the cell nuclei of tumor cells has been observed in several cancer types [41,61,62,63]. In the nucleus, NHERF-1 binding partners identified so far are β-catenin and TCF-1B, a transcription factor that associates with β-catenin and mediates its EMT transcriptional activity [41,62]. We have observed that MINDIN induces translocation of NHERF-1 towards the cytoplasmic and perinuclear compartments, but we have not observed NHERF-1 localization in the cell nucleus. Thus, our data suggest that MINDIN could enhance the activation of the Wnt/β-catenin pathway in prostate cancer cells by promoting a decrease in NHERF-1 expression at the plasma membrane, therefore releasing the membranous fraction of β-catenin. Then, this fraction would be available to translocate into the cell nucleus to promote β-catenin-dependent transcriptional activities. Whether NHERF-1 translocates to the nucleus with β-catenin in any stage during prostate cancer progression would require further study.

Our data show that MINDIN promotes the expression of osteoclastic- and osteoblastic-related genes that have been associated with prostate tumor progression and development of bone metastases [8,18,19]. However, overexpression of NHERF-1 caused no effect in most of the bone-related genes tested, suggesting that MINDIN induces osteomimicry by NHERF-1-independent mechanisms. Intriguingly, both MINDIN and NHERF-1 overexpression seem to increase RANKL expression in prostate cancer cells. MINDIN may affect RANKL expression by a NHERF-1 independent mechanism whereas it is possible that overexpression of NHERF-1 upon a certain high threshold could also trigger an increase in RANKL. A previous study described decreased osteoclast function in NHERF-1 knock out mice by a mechanism probably dependent on RANKL impaired production by NHERF-1-knocked out osteoblasts [47].

We have also observed that MINDIN triggers tumor cell proliferation, migration and adhesion in prostate adenocarcinoma TRAMP-C1 cells. In this regard, previous studies have reported that MINDIN activates NF-kappa β in colon cells [64]—a signaling pathway that has been shown to induce cell proliferation in prostate cancer cells [65]. NHERF-1 has been associated with regulation of NF-kappa β activation in inflammation processes [66]. Therefore, it might be possible that NHERF-1 mediates, at least in part, MINDIN-dependent action on prostate tumor cell proliferation via NF-kappa β activation.

A role of MINDIN in promotion of adhesion and outgrowth of hippocampal embryonic neurons has also been proposed by an unknown mechanism [67]. In addition, MINDIN–integrin interactions have been reported to be critical for neutrophil and macrophage adhesion and recruitment in in vivo inflammatory models [68]. In these studies, it was proposed that MINDIN bound to the extracellular matrix acted as an integrin ligand that enhanced cell adhesion and migration [68]. In this regard, our data show that NHERF-1 overexpression inhibits MINDIN-dependent actions on prostate cancer cell migration without affecting cell adhesion. Thus, although it is unlikely that NHERF-1 modulates extracellular binding interactions of MINDIN with integrins, a role of NHERF-1 on the regulation of intracellular responses triggered by MINDIN–integrin interactions could be feasible.

The present data collectively show that NHERF-1 is downregulated by MINDIN in primary prostate tumors causing an increase in tumor cell proliferation and migration.

## 4. Materials and Methods

### 4.1. Human Tissue Specimens

Primary prostate cancer samples (a total of 51) were collected from patients who had undergone radical prostatectomy at HM Sanchinarro Universitary Hospital (Madrid, Spain). The study project was approved by the Ethics and Clinical Trials Committee (CEIC) of HM Group Hospitals and the patients included in the study accepted the terms of the patient information document approved and provided by the Ethics Committee. In addition, 22 control prostate specimens without hyperplasia were collected from healthy deceased subjects following multiorganic recovery at Princesa Universitary Hospital (Madrid, Spain) (Ethical code: HM 12.04. 297-GHM; Approval date: 16 May 2012). Prostate extraction was performed after obtaining informed consent from the relatives of the deceased patients. Usage of clinical samples was authorized by HM Group and Princesa Hospital Ethics and Clinical Research Committee. Tissue samples were fixed in formaldehyde and subsequently embedded in paraffin for immunohistochemistry assessment. Clinical data from human samples were compiled in an adenocarcinoma sample database following anatomopathological criteria, including the admission number, age, PSA levels, Gleason score, D`Amico risk, TNM pathologic staging, perineural invasion and surgical margins (Table S1).

### 4.2. Animal Model

A C57BL/6 mouse (Charles River Laboratories, Wilmington, MA, USA) model of prostate cancer based on the implantation of prostate adenocarcinoma TRAMP-C1 cells was used as previously described [18,19]. The TRAMP model of prostate cancer induced in immunocompetent mice have detectable prostate tumors at 4 weeks after injection and can develop metastasis to different organs, which is a useful model to study prostate cancer progression [69]. TRAMP-C1 cells in the in vivo model were silenced with 3 specific siRNAs targeted to MINDIN (s97640;s97638;s87252) (Life Technologies, Paisley, UK). A scrambled sequence (control siRNA-A, Santa Cruz Biotechnology, Dallas, TX, USA) was used as a negative control. After 1 month, primary tumors were detectable and were extracted and stored in Trizol (Thermo Fisher Scientific, Waltham, MA, USA) for real time PCR analysis. Experimental protocols were approved by the Institutional Animal Care and Use Committee of San Pablo CEU University.

### 4.3. Cell Culture

Mouse adenocarcinoma prostate TRAMP-C1 (obtained from ATCC, Manassas, VA, USA: CRL-2730) cells were grown in DMEM supplemented with 5% FBS and 5% Nu-serum IV, 0.005 mg/mL bovine insulin and 10 nM dehydroxiandrosterone. Human prostate carcinoma cells LNCaP (obtained from ATCC: CRL-1740) were grown in RPMI-1640 supplemented with 10% FBS. Both cell lines were cultured with penicillin (100 units/mL) and streptomycin (100 µg/mL) in a 5% CO2 humidified incubator at 37 °C. TRAMP-C1 and LNCaP cells were incubated with 5 ng/mL MINDIN (R&D Systems, Minneapolis, MN, USA) for 6 or 24 h, when appropriate. It has been previously described that the medically relevant domain of MINDIN to discern healthy patients from prostate cancer patients by assessing serum levels of MINDIN is established between 1 and 10 ng/mL [16,70].

### 4.4. Immunohistochemistry and Immunofluorescence

Immunochemistry analyses were performed on 3 μm paraffin embedded tissue sections. Tissue sections were deparaffinized and endogenous peroxidase activity was quenched with 3% H_2_O_2_ (VWR, Fontenay-sous-Bois, France) in water for 30 min in the dark. Antigen retrieval was performed using 10 mM citrate pH 6 for 30 min. Following, unspecific interactions were blocked with serum blocking solution (Histostain-SP Broad Spectrum HRP: Life Technologies, Frederick, MD, USA) for 1 h and sections were incubated with a rabbit polyclonal NHERF-1 primary antibody (dilution 1/500) (Abcam, Cambridge, UK) or rabbit polyclonal MINDIN primary antibody (dilution 1/100) (R&D Systems, Minneapolis, MN, USA) overnight at 4 °C. After 24 h, samples were incubated for 1 h at room temperature with a biotinylated secondary antirabbit antibody and HRP-Streptavidin: Histostain-SP Broad Spectrum HRP (Horse Radish Peroxidase) (Life Technologies, Frederick, MD, USA) or with a secondary antirabbit antibody conjugated with HRP (dilution 1/200) (Santa Cruz Biotechnology). After washing, slides were incubated with DAB (3,3-diaminobenzidine, Life Technologies) and counter stained with hematoxylin. Absence of primary antibody was used as a negative control. Samples were mounted with DPX Mountant (VWR) and examined using a Leica (Wetzlar, Germany) DFC 425 camera connected to a Leica 5500B microscope.

Immunofluorescence assays were performed in cells fixed with 4% *p*-formaldehyde and permeabilized using 0.1% Triton in phosphate-buffered saline (PBS), pH 7.4. Nonspecific binding was blocked with 5% goat serum in bovine serum albumin (BSA), followed by overnight incubation with rabbit polyclonal NHERF-1 primary antibody (dilution 1/500) (Abcam). Cells were rinsed three times with PBS before incubation for 1 h with Alexa fluor 568-conjugated antirabbit IgG secondary antibody (Invitrogen, Waltham, MA, USA), respectively. Cell nuclei were stained with 4′,6-diamidino-2-phenylindole. Samples were mounted in FluorSafe Reagent (Calbiochem, La Jolla, CA, USA) and examined using a Leica DM 5500B microscope or Leica Stellaris SP5 confocal microscope.

### 4.5. Cell Silencing and Transfection

TRAMP-C1 cells were silenced with a mixture of three siRNAs (each at 20 nM) against different coding sequences of mouse MINDIN (s97640;s97638;s87252; Life Technologies, Paisley, UK) using lipofectamine RNAiMax (Life Technologies) overnight at 37 °C, following the manufacturer’s instructions. A scrambled sequence (control siRNA-A, Santa Cruz Biotechnology, Dallas, TX, USA) was used as a negative control for evaluating RNAi off-targeted effects, and in order to verify the accuracy of gene-specific siRNA-dependent changes in different parameters evaluated. Efficiency of MINDIN silencing was 85% after 48 h of transfection and 60% up to 15 days after transfection [18]. Efficiency of MINDIN silencing was assessed by real time PCR.

TRAMP-C1 cells were transfected with 2 μg of ^GFP^NHERF-1 (generously donated by Peter Friedman, Department of Pharmacology and Chemical Biology, University of Pittsburgh, PA, USA) using lipofectamine 2000 (Life Technologies) for 48 h at 37 °C, following the manufacturer´s instructions. As a negative control, 2 μg of pcDNA3.1 empty vector (generously donated by Peter Friedman) was used.

### 4.6. Western Blot Analysis

Total cell protein extracts were obtained with RIPA Buffer, supplemented with protease inhibitor cocktail (Sigma-Aldrich St. Louis, MO, USA), and phosphatase inhibitor cocktail Set II (Calbiochem). Western blot was performed as previously described [19] using a rabbit polyclonal NHERF-1 primary antibody (dilution 1/2000) (Abcam). α-tubulin (Sigma-Aldrich) was used as a loading control.

### 4.7. Real Time PCR

Total RNA was isolated from mouse prostate samples or from TRAMP-C1 cells by a standard procedure (Trizol, Life Technologies). Then, 2 µg of this RNA was retrotranscripted with a cDNA high capacity retrotranscription kit (Applied Biosystems, Grand Island, NY, USA) following manufacturer´s instructions. Gene expression was analyzed by real time PCR using Sybr premix ex Taq (Takara, Otsu, Japan) and an ABI PRISM 7500 system (Applied Biosystems). Mouse-specific primers were used (Table 1)

Alternatively, real time PCR was performed using predeveloped fluorogenic mouse-specific TaqMan MGB probes for MINDIN (Mm00513596_m1) (Life technologies). The relative gene expression in cell assays was represented as previously described [18,19]. Amplicon specificity was confirmed as the presence of a single peak in the melting curve for each qPCR reaction.

### 4.8. Proliferation, Migration and Adhesion Assays

The number of viable TRAMP-C1 cells was evaluated by a trypan blue exclusion assay as previously described [74]. TRAMP-C1 cell migration was assessed using an in vitro scratch assay in serum free medium as previously reported [75].

The adhesion of TRAMP-C1 adenocarcinoma cells to collagen surfaces was assessed by seeding calcein-AM- labeled TRAMP-C1 cells in a 6-well plate with collagen. TRAMP-C1 cells were preincubated with 2 μM calcein-AM (Thermo Fisher Scientific, Waltham, MA, USA) for 30 min. Next, the cells were washed with PBS and seeded onto collagen covered well surfaces. Nonadherent cells were removed after 30 min of incubation with complete medium followed by plate washing with PBS. Adherent cells were fixed with 4% paraformaldehyde. Images were obtained with an epifluorescence microscope (Leica DM5500B). The number of fluorescence-labeled cells was counted in 10 different fields per condition.

### 4.9. Statistical Analysis

All results are expressed as means ± SEM. Differences among conditions were evaluated by nonparametric variance analysis (Kruskal–Wallis) followed by Dunn’s test. Mann–Whitney test was performed to analyze the differences between control or scrambled siRNA samples and MINDIN-stimulated, MINDIN siRNA-transfected, NHERF-1, NHERF-1 MINDIN-stimulated, PCDNA 3.1 or PCDNA 3.1 MINDIN-stimulated transfected samples.

## 5. Conclusions

MINDIN promotes prostate cancer cell migration and proliferation via downregulation of NHERF1 levels. We propose that NHERF-1 downregulation by MINDIN has a key role during prostate cancer progression. This pathway could be a potential target to treat advanced prostate cancer.

## Figures and Tables

**Table 1:** Specific mouse primers used to perform quantitative PCR in prostate mouse samples and cell cultures.

Primer [Reference]	Forward (5′--->3′)	Reverse (5′--->3′)
Na^+^/H^+^ exchanger regulatory factor or NHERF-1 (NHERF-1) [47]	TCGGGGTTGTTGGCTGGAGAC	GAGCTCGCGCAAGTGGCTCT
Tartrate-resistant acid phosphatase (TRAP) [18,19]	CACGAGAGTCCTGCTTGTC	AGTTGGTGTGGGCATACTTC
Osterix [18,19]	CTGCCTGACTCCTTGGGACC	GCCATAGTGAGCTTCTTCCTCAA
RANK [18,71]	GCAACCTCCAGTCAGCA	GAAGTCACAGCCCTCAGAATC
Receptor activator of nuclear factor kappa-Β ligand (RANKL) [18,72]	TGTACTTTCGAGCGCAGATG	AGGCTTGTTTCATCCTCCTG
Osteoprotegerin (OPG) [18,73]	CAGAGCGAAACACAGTTTG	CACACAGGGTGACATCTATTC
Osteocalcin [18,73]	GCAATAAGGTAGTGAACAGACTCC	CCATAGATGCGTTTGTAGGCGG
Alkaline phosphatase [18,73]	CCAGAAAGACACCTTGACTGTGG	TCTTGTCCGTGTCGCTCACCAT
Runt-related transcription factor 2 (RUNX2) [18,73]	CCTGAACTCTGCACCAAGTCCT	TCATCTGGCTCAGATAGGAGGG
Beta Actin [73]	GAACCCTAAGGCCAACCGTG	ACCAGAGGCATACAGGGACAG

## Data Availability

The data presented in this study are available on request from the corresponding author. The data are not publicly available due to ethical reasons.

## Supplementary Materials

The following are available online at www.mdpi.com/xxx/s1,Figure S1, Whole blots (uncropped blots) showing that MINDIN decreases NHERF-1 expression in TRAMP-C1 prostate adenocarcinoma cells. TRAMP-C1 and LNCaP prostate cancer cells were stimulated with 5 ng/mL MINDIN for 24 h, Figure S2: ^GFP^NHERF-1 overexpression. Overexpression of NHERF-1 in TRAMP-C1 cells was achieved by transient transfection using 2 µg/mL of a ^GFP^NHERF-1 plasmid construct, Table S1: Clinical details of male patients diagnosed with prostate cancer.
