# Supplementary material for: MINDIN Exerts Protumorigenic Actions on Primary Prostate Tumors via Downregulation of the Scaffold Protein NHERF-1
Source: Cancers (Basel). 2021 Jan 24;13(3):436. doi: 10.3390/cancers13030436 (PMC7865820; doi:10.3390/cancers13030436)
Supplement: Supplementary file 1 [file cancers-13-00436-s001.pdf]

cancers

Article

MINDIN Exerts Pro-Tumorigenic Actions on Primary Prostate Tumors Via Downregulation of the Scaffold Protein NHERF-1

Luis Álvarez-Carrión, Irene Gutiérrez-Rojas, M. Rosario Rodríguez-Ramos, Juan A. Ardura, and Verónica Alonso

Supplementary Materials

Table S1. Clinical details of male patients diagnosed with prostate cancer.

Admission Number	Age	PSA ng/mL	Gleason Score	D'Amico Risk	TNM Pathologic Staging	Perineural Invasion	Surgical Margins
S12-6445-A8	63	7.4	3+4	INTERMEDIATE	pT2c	0	Negative
S12-6582-A14	60	14.0	3+3	INTERMEDIATE	pT2c	0	Negative
S12-6655-A7	63	12.0	4+3	INTERMEDIATE	pT2c	1	Negative
S12-6921-A13	69	8.6	3+3	LOW	pT2c	0	Negative
S12-7016-A1	67	5.9	4+3	LOW	pT2c	0	Negative
S12-7240-A1	62	8.8	3+3	LOW	pT2c	0	Negative
S12-7483-A1	53	9.0	3+3	LOW	pT2a	0	Negative
S12-8429-A1	67	6.5	3+3	LOW	pT2c	0	Negative
S12-8884-A19	65	12.6	3+4	INTERMEDIATE	pT3b	0	Negative
S12-9815-A8	50	6.8	3+4	INTERMEDIATE	pT2a	0	Negative
S12-10817-A4	71	8.8	4+5	HIGH	pT2b	0	Negative
S13-00299-A11	63	5.7	3+3	LOW	pT2c	0	Positive
S13-00601-A8	65	4.6	3+3	LOW	pT2a	0	Negative
S13-00839-A25	60	6.3	4+3	HIGH	pT3a	0	Positive
S13-01132-A80	60	ND	4+3	HIGH	pT3a	0	Positive
S13-01126-A11	61	11.0	3+3	INTERMEDIATE	pT2c	0	Positive
S13-03877-A11	52	7.0	3+3	INTERMEDIATE	pT2c	0	Positive
S13-04240-A14	64	9.0	3+4	INTERMEDIATE	pT2c	0	Positive
S13-04367-A22	41	4.4	3+3	LOW	pT2c	0	Positive
S13-05208-A14	62	10.0	3+4	INTERMEDIATE	pT3a	0	Negative
S13-06056-A1	70	7.9	3+4	INTERMEDIATE	pT2c	1	Negative
S13-07645-A14	55	4.7	3+4	INTERMEDIATE	pT2c	0	Negative
S13-07881-A2	62	10.0	3+4	INTERMEDIATE	pT2c	1	Positive
S13-05757-A22	64	7.0	3+3	LOW	pT3a	0	Positive
S14-00330-A46	54	5.0	3+3	LOW	pT3a	1	Negative
S14-00305-A10	58	12.5	3+4	INTERMEDIATE	pT2c	1	Negative
S14-00608-A34	65	6.4	3+3	LOW	pT2c	0	Negative
S14-00898-A13	61	3.4	4+3	LOW	pT3a	1	Negative
S14-01471-A11	67	4.5	3+4	INTERMEDIATE	pT2c	0	Negative
S14-02065-A8	66	7.0	3+4	LOW	pT2c	0	Negative
S14-03706 A23	72	23.4	4+3	HIGH	pT3a	0	Negative
S14-04255 A20	62	4.7	3+3	LOW	pT2c	0	Negative
S14-04561 A11	52	5.8	3+3	INTERMEDIATE	pT2b	0	Negative
S14-04586 A9	71	8.9	4+3	LOW	pT2c	0	Negative
S14-04742 A7	59	8.4	3+3	LOW	pT2a	0	Negative
S14-05759 A15	41	7.8	3+4	LOW	pT2c	0	Positive
S14-05813 A13	69	11.3	4+3	INTERMEDIATE	pT2c	0	Negative
S14-06003 A16	65	6.9	3+3	LOW	pT3a	0	Negative
S14-06114 A37	70	10.0	4+3	INTERMEDIATE	pT3a	0	Negative
S14-06637 A14	57	4.2	3+4	LOW	pT2c	0	Negative
S14-06642 A26	51	4.0	3+4	LOW	pT3a	0	Negative
S14-08126 A21	53	4.7	3+4	INTERMEDIATE	pT2c	0	Negative
S14-08224 A2	71	6.8	3+3	LOW	pT2c	0	Negative
S14-08142 A16	56	11.0	3+4	INTERMEDIATE	pT2c	0	Negative

**S14-08281 A8**	50	3.2	3+3	LOW	pT2c	0	Negative
**S14-09146 A2**	56	3.8	3+3	LOW	pT2a	0	Negative
**S14-09420 A12**	68	8.4	4+3	INTERMEDIATE	pT2c	0	Negative
**S14-09750 A13**	65	16.5	3+4	INTERMEDIATE	pT3a	0	Negative
**S14-09948 A22**	68	5.2	3+3	LOW	pT2c	0	Negative
**S14-10232 A20**	64	7.3	3+4	INTERMEDIATE	pT3a	1	Negative
**S14-10580 A11**	63	5.0	3+3	LOW	pT2c	0	Negative
**S14-10618 A1**	60	6.9	4+3	INTERMEDIATE	pT2c	1	Negative
**S14-10929 A9**	72	5.3	3+4	INTERMEDIATE	pT2a	1	Positive
**S14-11817 A12**	47	7.4	3+4	INTERMEDIATE	pT2c	0	Negative

(**A**)

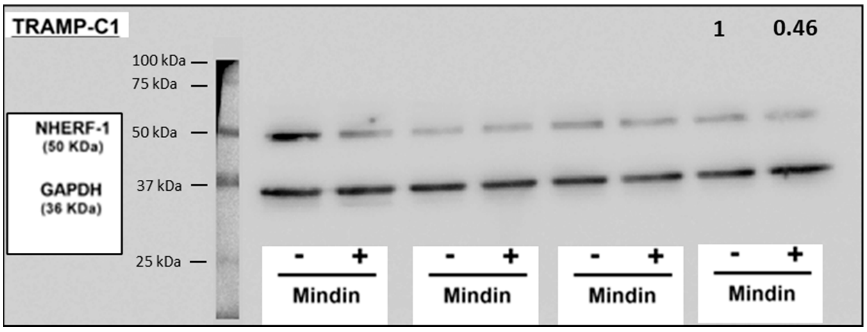

(B)

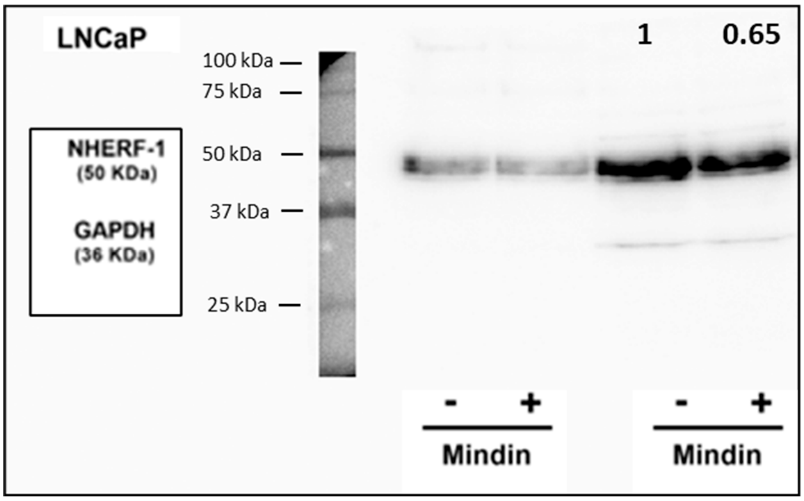

Figure S1. Whole blots (uncropped blots) showing that MINDIN decreases NHERF-1 expression in TRAMP-C1 prostate adenocarcinoma cells. TRAMP-C1 and LNCaP prostate cancer cells were stimulated with 5 ng/mL MINDIN for 24 h. NHERF-1 protein levels were assessed by western blot. Both NHERF1 (50 KDa) and GAPDH (36 KDa) bands are shown in representative western blot autoradiograms. Densitometric values of the bands represented in the manuscript are shown. All columns represent TRAMPC-1 (**A**) or LNCAP (**B**) cells stimulated (+) or not (−) with MINDIN.

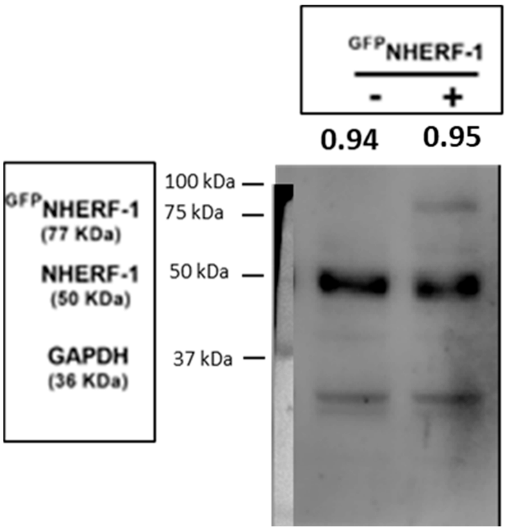

Figure S2. GFPNHERF-1 overexpression. Overexpression of NHERF-1 in TRAMP-C1 cells was achieved by transient transfection using 2 μg/mL of a GFPNHERF-1 plasmid construct. NHERF-1 protein expression was evaluated by western blot in GFPNHERF-1-transfected cells.
